# Knowledge and perception of hand hygiene: A survey using WHO standardized tools in Tehran, Iran

**DOI:** 10.22088/cjim.13.1.122

**Published:** 2022

**Authors:** Seyed Ali Dehghan Manshadi, Mojtaba Sedaghat, Fatemeh Mohammad Hashem, Mohammadreza Salehi, Esmaeil Mohammadnejad, Arash Seifi, Arezoo Salami Khaneshan

**Affiliations:** 1 Department of Infectious Diseases, Tehran University of Medical Sciences, Tehran, Iran; 2 Department of Community Medicine, Tehran University of Medical Sciences, Tehran, Iran; 3 Department of Internal Medicine, Tehran University of Medical Sciences, Tehran, Iran; 4 Department of Nursing and Midwifery, Tehran University of Medical Sciences, Tehran, Iran

**Keywords:** Hand hygiene, Knowledge, Perception, Cross infection

## Abstract

**Background:**

Proper hand hygiene is the most important action in preventing healthcare-associated infections (HCAIs). In this study, the knowledge and perception of hand hygiene assessed among nurses as the most exposed personnel to patients.

**Methods:**

In this analytical cross-sectional study, the nurses working in different wards of a collegiate tertiary hospital in Tehran were investigated by standardized WHO questionnaires.

**Results:**

Of the 101 participating nurses 89 (88.1%) were females. 81 (80.2%) had received formal related training. The 69 respondents estimated the mean prevalence of HCAI to be 38.91% and 98 (97.1%) considered hand hygiene an effective prevention in this regard. 78 (77.3%) perceived hand hygiene as the center priority; 82, 83 and 79 of participants would think that good hand hygiene matters for their superiors, colleagues and patients, respectively. The practice of hand hygiene was stated to be difficult by 48 (47.5%) respondents. There was no significant difference in self-reporting of hand hygiene practice among nurses in age (P=0.68), the degree of education (P=0.574), work experience (P=0.64), nor their wards (P=0.131). There was a significant reverse relationship with the supposed difficulty level of doing hand hygiene (P=0.049). The mean score of the nurses' knowledge was 66.53 (±9.41) based on the answers to the questions of the knowledge questionnaire.

**Conclusion:**

Knowledge and perception of hand hygiene, as this study showed, might not to be satisfactory; therefore, planning to improve these indicators and regular monitoring using standard tools is necessary for all healthcare centers.

Infection prevention and control (IPC) is a core component of patients’ safety program all around the world (1). Healthcare-associated infections (HCAIs) affect 1.4 million people worldwide each year (2). It has been estimated that 20 to 40 percent of HCAIs are preventable (3, 4). Appropriate hand hygiene during patient care is an important action for preventing and controlling infections (2). However, international compliance of hand hygiene among health care providers around the world is unacceptably low (5, 6). HCAIs lead to high mortality and cost in almost all countries especially in developing countries such as our country, Iran (7). Appropriate nurses’ hand hygiene has an important role in preventing HCAIs; therefore, the necessary information about hand hygiene should be provided for them. It is also known that the knowledge and perception of nurses about hand hygiene affect their performance (8).

WHO suggests a questionnaire to evaluate knowledge and perception about hand hygiene as the following categories: individual's perception about the risk of not practicing hand hygiene in relation to HCAI, individual’s perception about the control on his/her behavior resulting from the exerted pressure by a colleague (colleagues or higher officials). HCW knowledge monitoring is also possible with this organization's proposed questionnaire. The questionnaire also considers the examining of individual information about five hand hygiene situations and individual’s background information on different methods and their preferences relative to each other and the selected model in specific situations (9).

In this study, we reviewed the levels of knowledge and perception of nurses (who have the most contact with patients) in a tertiary referral collegiate hospital in Tehran, Iran. 

## Methods

In this analytical cross-sectional study, 101 nurses working in different wards of a tertiary referral collegiate hospital in Tehran were investigated. The judgmental sampling method was used for sampling. Different units of the hospital were considered as categories, and the samples (number of nurses) selected from each ward were based on the proportion of the nurses of that ward to the total nurses of the hospital. In this study, the evaluation tool was the questionnaire prepared and recommended by the World Health Organization (9). The questionnaire was translated to Persian language and localized to a better understanding by the participants. The questionnaire including demographic data such as age, sex, degree of education, profession, ward, and work experience; Knowledge part including questions about training courses, use of alcoholic handrub, source of HCAIs’ germs and transmission routes, hand hygiene methods and situations, time needed for proper handrub or hand wash; Perception survey was on the estimation of HCAI rate, effect of hand hygiene on HCAI prevention and patient outcome, priority of hand hygiene in the center, the effect of staff training and others. The participants who entered the study, were assessed by completing the questionnaire. This study was conducted in the emergency unit, ICU, NICU, internal medicine wards, infectious disease ward, pediatric and surgery units of a collegiate tertiary hospital in Tehran, during 2019.

**Statistical analysis**: The obtained results for the quantitative variables are in the form of mean and standard deviation (mean ± SD). Comparison among quantitative variable is done by t-test or in the presence of not normal distribution, it is done by Mann-Whitney U test. Comparison of qualitative has been performed by chi-square test or Fisher’s exact test. Correlation among the quantitative variables has been examined through Pearson correlation coefficient and Spearman rank correlation. In determining the differences of the indices in the presence of the basic characteristics of patients as confounding factors, multivariate logistic regression analysis was used and its results were stated as odds ratio (95% confidence interval). SPSS Version 21 and SAS Version 9.1 were used for statistical data analysis. The significance level was considered less than 0.05. 

**Research Ethics: **In this study no intervention was done, and no cost was imposed on the participants. Researchers, in all stages, adhered to the principles of the Helsinki manifesto and the Ethics Committee of Tehran University of Medical Sciences as medical student’s dissertation with referral No.32152. 

## Results

**General Data: **One hundred and one nurses participated in this study, included 12 (11.9%) men and 89 (88.1%) women. The mean age was 33.3 (±7.56) years. The mean of work experience was 9.05 (±6.8) years. 

**Figure 1:**
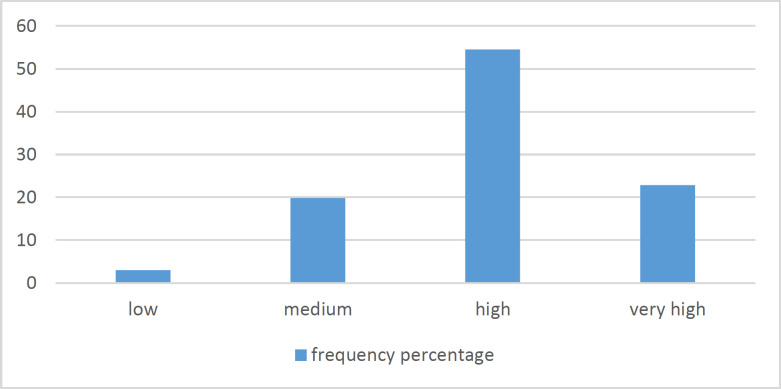
Priority of hand hygiene in this center in the nurses’ viewpoint

**Perception Survey:** Eighty one (80.2%) nurses told they were already trained for proper hand hygiene but 20 (19.8%) nurses claimed they were not trained formally before. Ninety six individuals (95%) used alcoholic handrub (AHR) as a routine practice. According to the nurses’ viewpoint, they estimated 38.91% (±18.90) of patients were affected by HCAIs. The effect of HCAIs on patients’ outcome was assumed very high (19.8%), high (68.3%), low (10.9%), and very low (1%) based on the nurses’ answers. Almost all nurses accepted the high effect of hand hygiene in prevention of HCAIs. The priority of hand hygiene in the hospital (expressed by nurses) showed in figure1. Performing proper hand hygiene assumed to be easy for 53 (52.5%) nurses and it is considered to be difficult for 48 (47.5%). The nurses thought hand hygiene compliance among nurses was 60.31% (±25.56). According to the nurses’ attitude, 81% of hospital authorities were concerned about the personnel’s hand hygiene. They thought the colleagues mattered 82.2% about their proper hand hygiene. The amount of importance given by patients to nurses’ proper hand hygiene was 78.2%. In overall, 76% (±17.6) of nurses claimed doing proper hand hygiene. 

**Figure 2:**
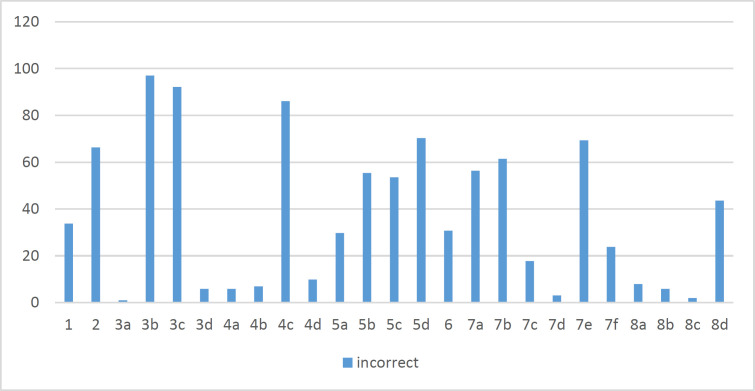
The number of incorrect answers to the questions of the knowledge questionnaire

**Knowledge Survey:** The mean score of nurses' knowledge was 66.53 (±9.41) based on the answers to the questions of the knowledge questionnaire (9). Figure 2 shows the status of the participants’ answers.

1. The main route of cross-transmission of potentially harmful germs between patients in a health-care facility.

2. The most frequent source of germs responsible for health care-associated infections.

3. Hand hygiene actions prevent transmission of germs to the patient. 3a: Before touching a patient. 3b: Immediately after a risk of body fluid exposure. 3c: After exposure to the immediate surroundings of a patient. 3d: Immediately before a clean/aseptic procedure.

4. Hand hygiene actions prevent transmission of germs to the health-care worker. 4a: After touching a patient. 4b: Immediately after a risk of body fluid exposure. 4c: Immediately before a clean/aseptic procedure. 4d: After exposure to the immediate surroundings of a patient.

5. Statements on alcohol-based handrub and handwashing with soap and water (True/False). 5a: Handrubbing is more rapid for hand cleansing than handwashing. 5b: Handrubbing causes skin dryness more than handwashing. 5c: Handrubbing is more effective against germs than handwashing. 5d: Handwashing and handrubbing are recommended to be performed in sequence.

6. Minimal time needed for alcohol-based handrub to kill most germs on the hands.

7. Hand hygiene method required in the situations. 7a: Before palpation of the abdomen. 7b: Before giving an injection. 7c: After emptying a bedpan. 7d: After removing examination gloves. 7e: After making a patient's bed. 7f: After visible exposure to blood.

8. Increased likelihood of colonization of hands. 8a: Wearing jewelry. 8b: Damaged skin. 8c: Artificial fingernails. 8d: Regular use of a hand cream.

**Hand Hygiene Self-reporting Analysis:** There was no significant difference in the self-reporting of the hand hygiene practice among nurses in different wards (P=0.131). There was no significant relationship between the self-reporting of the hand hygiene practice and the work experience and the age of the nurses (P=0.64 and P=0.68 respectively). There was no significant relationship between the self-reporting of the hand hygiene practice and the estimation of HCAIs rate by nurses (P=0.77). There was no significant relationship between the self-reporting of the hand hygiene practice and the degree of education (P=0.574). There was a significant reverse relationship between the self-reporting of the hand hygiene practice and the supposed difficulty level of doing proper hand hygiene (P=0.049). There was a significant direct relationship between the self-reporting of the hand hygiene practice and the real performance of hand hygiene (P=0.001).

## Discussion

The attitude of the staff was questioned in the first part of the perception questionnaire. They had a positive opinion about the effects of performing proper hand hygiene in preventing HCAIs (97.1%, high and very high affect), but the real hand hygiene compliance among the nurses had no relationship with their beliefs about HCAIs and the prevention with hand hygiene, this finding was opposed to Jenner and O'Boyle’s study and similar to Sax’s study (10-12). 

Among the evaluated factors, the more the hand hygiene was important for the hospital authorities, co-workers and patients, the more positive effect was on the participants’ hand hygiene practice. In Pessoa’ study, the hospital authority’s opinion (13), in Sax’s study, patient’s opinion (10) and in Jenner’s study, none of the subjective norms (11) was correlated with participants’ hand hygiene performance. Individual perception of difficulty of doing proper hand hygiene was examined during a questionnaire; this practice was not difficult on the viewpoint of 52.5% nurses and a reverse relationship was found. Similar results were obtained in the researches of Sax, Jenner and Pessoa (10, 11, 13). 

A point that should be considered is the difference and relationship between self-reporting and the observed performance. In the year 2000, O'Boyle assessed the theory of planned behavior in the field of hand hygiene, he showed a weak relationship between these two (r=0.201). Jenner, in 2005, as a result of his study, suggested that there was no significant relationship between these two (11). This difference (between self-reporting and real performance) to be of any reason, is important for these aspects: first, when an individual's perception of his/her performance is so different from the reality, his/her readiness for corrective programs will be low; Second, the effective factors that have been identified in our study (or other perception studies) can improve hand hygiene only if self-reporting is a good representative of actual function.

In the field of nurses’ knowledge, the mean of knowledge in this study was 66%. Compared to the quasi-empirical study of the year 2009 in which WHO strategy was used in several countries: it is more in Saudi Arabia and lower in Italy and Pakistan (14). Training of personnel for hand hygiene principles and retraining periodically is of utmost important to improve hand hygiene compliance in a care center. 

Due to the relationship between the perception of the difficulty and the self-reporting of hand hygiene compliance, there is a need for detection of external and internal obstacles via direct (field observation) and indirect (questionnaire surveys) assays. 

 In conclusion, Knowledge and perception of hand hygiene, as this study showed, might not to be satisfactory; therefore, planning to improve these indicators and regular monitoring with standard tools is necessary for all healthcare centers. Nurses' self-reported adherence was determined more by normative beliefs and control behavior than behavioral beliefs; although incomplete without the direct observation as a mean for monitoring true compliance; this study can form a basis for future promotional interventions.

## Funding:

This study was not funded.

## Conflict of interest:

Authors have no conflicts of interest to declare.
